# The expanding functions of thyroid hormone

**DOI:** 10.1186/s13578-017-0176-0

**Published:** 2017-10-19

**Authors:** Jiemin Wong, Shaochung Victor Hsia

**Affiliations:** 1 Shanghai Key Laboratory of Regulatory Biology, Fengxian District Central Hospital-ECNU Joint Center of Translational Medicine, Institute of Biomedical Sciences and School of Life Sciences, East China Normal University, Shanghai, 200241 China; 2 Department of Pharmaceutical Sciences, School of Pharmacy, University of Maryland Eastern Shore, Princess Anne, MD 21853 USA

Thyroid hormone (TH) exerts a pleiotropic action on most, if not all, cell types, ranging from the regulation of development, metabolism, and neuronal signaling [1–3]. More than 50 years ago Jamshed Tata first reported that the response to TH involved transcriptional changes in the cell [4]. It is clear now that this transcriptional effect, also termed genomic action, is primarily mediated by the nuclear thyroid hormone receptor (TR) [5]. Accumulative studies indicate that TR regulates target gene expression through a diverse group of accessory proteins collectively termed corepressors and coactivators.

Besides the genomic actions through TR, TH can also act through non-genomic pathways [6]. The non-genomic actions have been shown to involve binding of TH to membrane receptors, to membrane bound protein kinases, or to extranuclear TRs. For example, the rapid TH effect on cardiac cells appears to involve TR-mediated activation of phosphoinositide 3-kinase in the cytosol and binding of TH to the membrane receptor, integrin αVβ3, which in turn activates a MAPK signaling cascade [6].

Within the general themes of genomic and non-genomic actions of TH, the recent progresses in three new areas for TH actions are reviewed in this thematic series. First, Ying et al. sum up how TH regulates microRNA expression and how microRNAs can fine tune TH function in cardiac and skeletal muscle [7]. Second, Yen et al. focus on the function and molecular mechanisms by which TH regulates autophagy and mitochondrial turnover and the implications for non-alcoholic fatty liver disease (NAFLD) [8]. Third, Hsia et al. provides new insights on TH-mediated regulation of herpes virus infections through non-genomic action [9]. It is our sincere hope that this thematic series brings our readers some of the new breakthroughs and developments in the field of TH action.

## References

[1] Mullur R, Liu YY, Brent GA (2014). Thyroid hormone regulation of metabolism. Physiol Rev.

[2] Ng L, Kelley MW, Forrest D (2013). Making sense with thyroid hormone—the role of T(3) in auditory development. Nat Rev Endocrinol..

[3] Ortiga-Carvalho TM, Sidhaye AR, Wondisford FE (2014). Thyroid hormone receptors and resistance to thyroid hormone disorders. Nat Rev Endocrinol..

[4] Tata JR (2013). The road to nuclear receptors of thyroid hormone. Biochim Biophys Acta.

[5] Brent GA (2012). Mechanisms of thyroid hormone action. J Clin Invest..

[6] Davis PJ, Goglia F, Leonard JL (2016). Nongenomic actions of thyroid hormone. Nat Rev Endocrinol..

[7] Zhang D, Li Y, Liu S, Wang YC, Guo F, Zhai Q, Jiang J, Ying H (2017). microRNA and thyroid hormone signaling in cardiac and skeletal muscle. Cell Biosci.

[8] Sinha RA, Yen PM (2016). Thyroid hormone-mediated autophagy and mitochondrial turnover in NAFLD. Cell Biosci.

[9] Figliozzi RW, Chen F, Hsia SV (2017). New insights on thyroid hormone mediated regulation of herpesvirus infections. Cell Biosci.

